# Breaking the Silence: Psychological Abuse Among Patients with Breast Cancer

**DOI:** 10.3390/healthcare13222823

**Published:** 2025-11-07

**Authors:** Turki S. Alqurashi, Abrar I. Aljohani

**Affiliations:** 1 Department of Social Work, Al-Lith University College, Umm Al-Qura University, Makkah 28429, Saudi Arabia; tsmqurashi@uqu.edu.sa; 2 Department of Clinical Laboratory Sciences, College of Applied Medical Sciences, Taif University, Taif 21944, Saudi Arabia

**Keywords:** breast cancer, patients and survivors, psychological abuse, risk factors, intimate partner violence, IPV, cross-sectional, intimacy, marital life

## Abstract

**Background:** Research on psychological abuse among patients with breast cancer and survivors of breast cancer in Saudi Arabia is scarce. This study aimed to identify psychological abuse and its associated factors among these individuals. **Methods:** This cross-sectional study included 146 patients with breast cancer and survivors of breast cancer. Data were collected from December 2024 to April 2025 using a modified survey instrument based on the United Nations Economic Commission for Europe violence against women module. An online questionnaire comprised two parts: the first collected demographic characteristics, including age, education, employment, breast cancer diagnosis, and mastectomy duration, and the second assessed psychological abuse via four items: insults, belittlement/humiliation, intimidation, and undermining of relationship stability. The association between psychological abuse and sociodemographic factors was assessed using the chi-square test. Significant associations in bivariate analyses were subsequently analyzed using exploratory logistic regression. **Results:** Approximately 20.5% of participants reported experiencing at least one form of psychological abuse. The most commonly reported behaviors were insults and undermining of relationship stability (both 20.5%), followed by belittlement/humiliation (17.8%) and intimidation (15.1%). Bivariate analyses indicated a greater incidence of humiliation among women whose spouses were unemployed or retired, as well as among those with more than six children. Logistic analysis indicated that spouse unemployment or retirement (OR = 5.36, 95% CI 1.62–17.74, *p* = 0.006) and having more than six children (OR = 5.84, 95% CI 1.33–25.55, *p* = 0.019) were associated with belittlement/humiliation, even after FDR correction. No significant correlations were identified regarding patient age, education, mastectomy status, or duration since diagnosis. Model diagnostics demonstrated a satisfactory fit (Nagelkerke R^2^ = 0.22; accuracy = 82.2%) and a lack of multicollinearity (VIF = 1.00–1.03). **Conclusions:** Psychological abuse affects around 20% of women diagnosed with breast cancer, especially those with unemployed spouses and larger families. These results highlight the need for psychological screening and couple-based therapies in cancer care to mitigate marital stresses and enhance survivors’ well-being.

## 1. Introduction

Breast cancer is among the most common malignancies afflicting women globally. In addition to its serious physical health effects, the disease can have emotional and psychological well-being impacts on patients, such as anxiety, altered body image, strained interpersonal relationships, and worry of recurrence [1]. Recent global estimates underscore the increasing prevalence of breast cancer and the substantial influence it has on the quality of life of women, particularly in countries where screening and early detection programs are still in the process of evolving [2]. The prevalence of breast cancer in Saudi Arabia is rising significantly, making it the most commonly diagnosed cancer in the country [3]. Such growth underscores the urgent need for research that not only addresses clinical outcomes but also the psychosocial challenges that affected women encounter.

The physical weakness of women due to the disease and after surgical procedures may result in diminished sexual function, which might then increase their risk of intimate partner violence (IPV), including sexual and psychological abuse [4]. IPV is a form of interpersonal violence that is more prevalent than domestic violence [5,6]. The World Health Organization defines IPV as any conduct within an intimate relationship that results in physical, psychological, or sexual harm to the individuals involved [7]. Psychological abuse is a systematic approach of verbal and emotional manipulation, humiliation, control, or coercion that can profoundly affect an individual’s mental well-being and may be caused by social isolation, diminished self-esteem, and compromised decision-making abilities [8]. Recently, researchers have examined the correlation between IPV and disease burden in women, including those with breast cancer [9]. IPV concurrently affects an estimated 12.5% of patients with breast cancer. The underreporting of IPV likely leads to an underestimation of this estimate [10,11]. A cancer diagnosis can serve as a catalyst for abuse, as reported by one woman who endured emotional abuse during her 15-year marriage, with an observed escalation following her diagnosis [12,13]. One woman reported that her husband’s control over her intensified following her breast cancer diagnosis [14]. Therefore, the incidence of IPV among women with cancer is twice that of women without cancer. Thus, women who have encountered IPV may find it even more challenging to cope with and recover from a cancer diagnosis. The underreporting of IPV hinders efforts to understand its actual burden and formulate effective prevention strategies. Psychological abuse is one of the most common but least reported forms of IPV. Women diagnosed with chronic and life-threatening diseases, such as breast cancer, may particularly suffer from psychological abuse. The stress associated with a diagnosis, prolonged treatment, and changes in family or marital dynamics can intensify relational conflicts and heighten dependency. In this context, IPV may ultimately compromise health outcomes and overall survivorship, potentially increasing psychological distress, delaying treatment decisions, or hindering recovery [15,16,17,18,19].

Psychological (emotional) abuse is also the most common form of IPV in Saudi Arabia [20] and the most common form of abuse among women with cancer [21]. The scarcity of data regarding the correlation between IPV, particularly psychological abuse, and breast cancer in Saudi women has presented significant obstacles for researchers and practitioners. A lack of evidence hinders researchers’ ability to identify risk factors, compare findings with international data, and develop culturally pertinent screening tools. In the absence of local data, practitioners encounter difficulties in enhancing awareness, implementing systematic screening, and delivering tailored psychological support to affected patients. Therefore, it is imperative to conduct a study that assesses psychological abuse among patients with breast cancer and breast cancer survivors. Thus, this study aimed to ascertain the psychological abuse and the factors contributing to it among patients with breast cancer and breast cancer survivors in Saudi Arabia using a cross-sectional survey. The survey evaluates the prevalence of psychological abuse and its correlation with factors like age, education, partner’s work status, number of children, and cancer-related clinical features. We hypothesized that psychological abuse is common among breast cancer patients and that its frequency is correlated with specific sociodemographic factors.

## 2. Materials and Methods

### 2.1. Participants

This cross-sectional study employed a convenience sampling approach, primarily including all Saudi women aged >18 years, regardless of marital status (married, divorced, or widowed), who were diagnosed with breast cancer. Both current patients and survivors who completed therapy were included. The study omitted a cutoff age to increase the number of Saudi women eligible to participate. This study excluded women who were not Saudi nationals, single, and with other types of cancer, as well as men. As the pattern of missingness was random and did not affect the sample characteristics, cases with missing data on core IPV or sociodemographic variables (<5%) were excluded using listwise deletion. The anticipated number of participants was estimated using the Raosoft calculator for sample size, considering a 95% confidence level, a 5% sampling error, and a 50% prevalence of the result of interest. Employing a 50% response distribution necessitates a larger sample size while preserving sufficient power and variability. Thus, we aimed to recruit at least 377 patients with breast cancer and breast cancer survivors. However, only 146 were recruited within the established timetable. Recruiting patients with breast cancer in Saudi Arabia, especially those willing to disclose experiences of IPV, is fundamentally difficult due to cultural sensitivities, stigma associated with both the disease and gender-based violence, and restricted access to healthcare environments. There is ample evidence in the literature highlighting these challenges. Studies have reported sample sizes in Saudi Arabia as low as 51 and 145 [22,23], suggesting that even smaller samples have shed light on psychosocial outcomes and quality of life for Saudi patients with breast cancer.

The study population consisted mainly of females diagnosed with breast cancer, as per the inclusion criteria. Participants’ ages ranged from 18 to over 50 years. Their sociodemographic characteristics are thoroughly summarized in Table 1. More participants were aged 36–49 years (n = 94, 64.4%). Concerning the educational background of the participants and their partners, most possessed a secondary school diploma (n = 72 [49.3%] and 90 [61.16%], respectively). Among the participants, 84 (57.5%) were unemployed, while 66 (45.2%) of their partners were employed. In addition, 132 (90.4%) participants had been married for over 6 years. The age difference between the participant and their partners was mainly 5–9 years (n = 48, 32.9%). Regarding family size, 76 (52.1%) participants reported having 4–6 children, while 46 (31.5%) reported having ≤3 children. Finally, 70 (47.9%) had been diagnosed with breast cancer for over 1 year, and 80 (54.7%) had undergone a mastectomy.

### 2.2. Instrument

Data were collected using items modified from the survey module on violence against women developed for the United Nations Economic Commission for Europe [24]. Considering the cultural context of Saudi Arabia, the initial survey items may be unsuitable for studies in this country. Thus, it would be inappropriate to use the universally accepted IPV survey module in Saudi Arabia [20]. Consequently, the questionnaire was specifically tailored for Saudi culture [24]. This approach aimed to minimize ascertainment bias and enable the assessment of the differing prevalence and consequences of IPV categories to ensure accurate data collection.

To evaluate clarity and contextual appropriateness, the adaptation process followed established cultural validation procedures: (1) forward–backward translation by bilingual experts, (2) review by an expert panel in public health and social sciences for semantic and conceptual equivalence, and (3) pilot cognitive testing with 12 Saudi women. To ensure cultural relevance, minor linguistic adjustments were made. The recall period for all IPV items referred to experiences that occurred within the previous 12 months. To facilitate disclosure, reduce respondent burden, and minimize subjective variation in interpreting severity levels, a binary (yes/no) response format was used. This methodology is consistent with the IPV screening instruments that had been used and was appropriate for the study’s objective of estimating prevalence and identifying associated factors, rather than measuring degree of severity gradients. A participant was classified as having experienced psychological IPV if she answered “Yes” to one or more items in this subscale.

The assessment of internal consistency using Cronbach’s alpha demonstrated satisfactory reliability (α = 0.85). Item homogeneity was indicated by corrected item–total correlations ranging from 0.70 to 0.92. The factor loadings obtained from principal axis factorization were 0.57 to 0.85, suggesting that all items made an important contribution to the single latent construct. Table 2 shows that all items are loaded on the same latent factor, as evidenced by communalities ranging from 0.57 to 0.85.

The final questionnaire comprised two parts. The first part collected demographic characteristics, including age, educational attainment, work position, marital status, and details about the length of time since breast cancer diagnosis and subsequent mastectomy. Investigating the educational and professional backgrounds of the participants’ spouses was essential. The second part aimed to assess experiences of psychological abuse, using a four-item questionnaire asking about verbal insults, belittlement/humiliation, intimidation (e.g., threats or controlling behaviors), and the undermining of relationship stability by their spouse in the previous 12 months. Table 2 shows the specific questions used to assess psychological abuse.

### 2.3. Procedure

This study collected data using an online survey and was conducted between December 2024 and April 2025. It employed online tools due to data from Global Media Insight for 2024 indicating that Saudi Arabia has 37.10 million internet users, representing 99% of the total population. Saudi Arabia also currently holds the distinction of having the largest social media presence globally [25]. Thus, the sample was first acquired using social media platforms, with the target audience addressed by sending Google Forms links to relevant Facebook, Telegram, WhatsApp, and Instagram profiles, such as cancer patient support groups. To increase the number of participants, URLs to Google Forms were sent via email and WhatsApp to 18 Saudi cancer groups and charities. These connections supplied individuals who expressed a desire to participate in this study with pertinent study-related information (an explanation of the nature of the study), ensuring informed consent, and then provided those who consented to participate with the questionnaire instrument.

### 2.4. Ethical Considerations

Before initiating the data collection phase, ethical approval was obtained from the Biomedical Research Ethics Committee of Umm Al-Qura University (approval no. HAPO-02-K-012-2024-12-2416). It is essential to evaluate ethical implications throughout the research process to minimize any potential harm. Following these discussions, the participants received non-intrusive questions concerning psychological abuse. They were thoroughly informed about the study’s objectives, and their participation in this study was entirely voluntary. It was also confirmed that they faced no personal, social, or cultural pressures related to their participation. The data collected during this study were only accessible to the principal author, and all participant information was handled with the highest level of confidentiality. This study is presented in accordance with the STROBE reporting checklist.

### 2.5. Data Analysis

Statistical analyses were conducted using SPSS Statistics, version 24 (IBM Corp., Armonk, NY, USA). The internal consistency and dimensionality of the psychological abuse items were assessed before analysis using item–total correlations, while validity was evaluated through factor loadings, which were calculated via principal axis analysis. Cronbach’s α was used to assess internal reliability. Since all four questions clustered on a single factor, a composite binary variable (“any psychological abuse”) was established to represent the occurrence of at least one affirmative (“Yes”) answer.

Participant characteristics and psychological abuse prevalence were summarized using descriptive statistics (frequencies and percentages). Four aspects of psychological abuse were assessed: insults, belittlement/humiliation, intimidation, and undermining of relationship instability. Pearson’s chi-square tests were used to evaluate each form in bivariate analyses. Fisher’s Exact Test (two-tailed) was used when the expected counts of more than 20% of cells were less than five or when any cell had an expected count of zero. To account for multiple comparisons, *p*-values were adjusted using the Benjamini–Hochberg false discovery rate (FDR) method (*q* < 0.05). Despite minor differences among subgroups, belittlement/humiliation was the only variable that exhibited statistical significance. These are clinically meaningful manifestations of emotional IPV, frequently associated with distress, decreased self-esteem, and disrupted relationships. This domain was selected for multivariable modeling to safeguard the stability of estimates related to low-frequency IPV subtypes and to ensure both clinical significance and statistical clarity. The dependent variable in the multivariable model was belittlement/humiliation, chosen for its higher variability and sufficient positive cases (*n* = 26). In this model, 1 indicates that the variable is present, while 0 indicates its absence. Model feasibility and conceptual relevance were the primary factors influencing this decision, rather than post hoc statistical significance. The remaining items were not modeled in multivariable analyses due to their low prevalence and nonsignificant bivariate associations, which would likely have yielded uninterpretable or unstable estimates. To improve the stability of the model, predictors with sparse categories were converted into binary variables. Findings from the multivariable logistic regression were presented as odds ratios (ORs) with 95% confidence intervals (CIs). Statistical significance was set at *p* < 0.05.

Multicollinearity was assessed using the Variance Inflation Factor (VIF < 2.0). The model’s fit was evaluated using the Hosmer–Lemeshow statistic and Nagelkerke R^2^. Due to the low outcome number, a Firth penalized logistic regression was conducted to evaluate robustness.

The logistic regression models employed OR with 95% confidence intervals as effect size measures, while Cramer’s V was employed to calculate the effect size for chi-square tests. In the regression models, missing data was handled through listwise deletion, which involved the exclusion of cases with missing values in any of the included variables from the analysis.

A directed acyclic graph (DAG), constructed in DAGitty (https://www.dagitty.net/dags.html, accessed on 15 September 2025) to demonstrate the hypothesized associations among clinical factors (time since diagnosis, mastectomy), partner-related factors (education, employment, age difference), and marital/family factors (duration of marriage, number of children), guided covariate selection. The DAG was created by considering clinical plausibility and published literature. DAGs have been more frequently recommended in observational research as a means of identifying confounders, preventing inappropriate adjustments, and enhancing causal interpretability. Using DAGitty, the graph was generated and analyzed to estimate the association while minimizing bias from confounding. This software provides a minimally sufficient adjustment set. Because the design of this study is cross-sectional, results are interpreted as associations.

## 3. Results

### 3.1. Psychological Abuse Among Patients with Breast Cancer

Among the 146 participants, 30 (20.5%) reported experiencing insults, a decline in self-worth, and instability in their relationships with their partners Table 3. Similarly, 26 (17.8%) reported experiencing belittlement and humiliation, while 22 (15.1%) reported they faced intimidation.

### 3.2. Distribution of Sociodemographic Among Participants Reporting Insults and Erosion of Self-Worth

Descriptive analyses of the participants who reported experiencing insults and a decrease in self-worth (n = 30, 20.5%) by various sociodemographic and clinical characteristics (Table 4) revealed that a considerable percentage had partners with low educational attainment (n = 22, 66.7%), while almost half had a partner who was retired (n = 14, 46.7%). Notably, a considerable percentage of these participants had been married for >6 years (n = 28, 93.3%). In addition, high percentage of these participants were not the same age as their partners (n = 24, 80.0%). Moreover, more of these participants had been diagnosed with breast cancer for >1 year (n = 20, 66.7%). Finally, more of these participants had undergone a mastectomy (n = 18, 60.0%; Table 4).

Although there were variations in the proportions of certain subgroups, these differences were not statistically significant (all *p* > 0.05) when we investigated the correlations between insults and a decrease in self-worth and a variety of sociodemographic characteristics, such as age, partner’s education, number of children, length of marriage, age difference with partner, time since diagnosis, and mastectomy status. Nevertheless, a limited number of significant correlations were identified. Patients who reported insults were more likely to have partners who were unemployed or retired (χ^2^ = 10.011, *p* = 0.017; Table 5). Following the implementation of the Benjamini–Hochberg FDR correction, no associations retained statistical significance, suggesting that the observed differences should be regarded with caution as exploratory results. The effect sizes for chi-square tests were small to moderate, as demonstrated by the Cramer’s V values (Table 5).

### 3.3. Distribution of Sociodemographic Among Participants Reporting Belittlement and Humiliation

Descriptive analyses of the participants who reported experiencing belittlement and humiliation (n = 26, 17.8%) by various sociodemographic and clinical characteristics (Table 6) revealed that a considerable percentage had partners with low educational attainment (n = 18, 69.2%), while almost half had a partner who was retired (n = 14, 53.8%). Notably, all of these participants had been married for >6 years (n = 26, 100%). In addition, most of these participants were not the same age as their partners (n = 22, 84.6%). Moreover, most of these participants had been diagnosed with breast cancer for >1 year (n = 20, 76.9%). Finally, just over two-thirds of these participants had undergone a mastectomy (n = 18, 69.2%; Table 6).

Although there were variations in the proportions of specific subgroups, these discrepancies were not statistically significant (all *p* > 0.05) when examining the correlations between belittlement and humiliation alongside various sociodemographic characteristics. Nevertheless, a few significant correlations were identified. Patients with unemployed or retired partners reported more belittlement and humiliation than those with employed or business partners (χ^2^ = 16.680, *p* < 0.001; Table 7). Additionally, patients with a spouse age difference of more than ten years reported experiencing belittlement and humiliation more frequently than those with smaller age disparities (χ^2^ = 11.891, *p* = 0.008; Table 7). Moreover, patients with more than six children reported higher belittlement and humiliation than those with fewer children (χ^2^ = 15.520, *p* = 0.003; Table 7). Similar associations were found after applying Benjamini–Hochberg FDR correction. Effect sizes varied from small to large across the examined variables, with the majority of associations exhibiting small to moderate strengths (Table 7).

In bivariate analysis, only humiliation had significant correlations to many demographic factors; hence, it had been selected as the dependent variable (1 = humiliation present, 0 = absence). To increase model stability with just 30 abuse instances, variables have been categorized into binary categories: age difference (<10 vs. ≥10 years) and number of children (≤6 vs. >6). Preliminary chi-square analysis revealed that unemployed and retired partners had a significantly higher incidence of abuse. To provide stable results in logistic regression, we collapsed employment status into a binary variable, classifying unemployed and retired individuals as the high-risk category (coded 1) and employed/business as the reference group (coded 0). Having more than six children was a significant predictor of abuse, with these patients approximately six times more likely to report psychological abuse than those with fewer children (OR = 5.84, 95% CI: 1.33–25.55, *p* = 0.019). Patients whose spouses were employed or in business were significantly less likely to report abuse than those whose partners were unemployed or retired. A larger age difference between spouses (≥10 years) correlated with increased risks of psychological abuse (OR = 2.20, 95% CI: 0.75–6.48), although this finding did not achieve statistical significance (*p* = 0.152; Table 8).

The robustness of associations was evaluated by retaining continuous variables for the age difference between partners and the number of children in this exploratory model. The employment status of partners was examined as a four-level categorical variable (1 = employed, 2 = retired, 3 = unemployed, 4 = business/self-employed), with the employed category as the reference group. Both retired and unemployed spouses were significantly associated with increased likelihood of belittlement/humiliation when employment was modeled in its full categorical structure (employed = reference). Participants with retired partners had odds 4.7 times higher (OR = 4.74, 95% CI 1.39–16.17, *p* = 0.013), while those with unemployed partners had odds nearly seven times higher (OR = 6.91, 95% CI 1.72–27.79, *p* = 0.006). The unstable estimation (no events) resulted from the limited number of participants whose partners were in business. The number of children and the age difference between the partners showed positive but nonsignificant associations (Table 9).

The model’s predicted probabilities (margins) revealed that the risk of reporting belittlement/humiliation was approximately 6% for participants with employed partners, 28% for those with retired partners, and 36% for those with unemployed partners. These predicted probabilities clarify the absolute differences underlying the ORs reported in Table 9.

### 3.4. Distribution of Sociodemographic Among Participants Reporting Intimidation

Descriptive analyses of the participants who reported experiencing intimidation (n = 22, 15.1%) by various sociodemographic and clinical characteristics (Table 4) revealed that a considerable percentage had partners with low educational attainment (n = 16, 72.7%), while almost half had a partner who was retired (n = 10, 45.5%). In addition, most of these participants had been married for >6 years (n = 18, 81.8%). In addition, most of these participants were not the same age as their partners (n = 18, 81.8%). Moreover, most of these participants had been diagnosed with breast cancer for >1 year (n = 16, 72.7%). Finally, most of these participants had undergone a mastectomy (n = 14, 63.6%; Table 10).

Although there were variations in the proportions of specific groups, these differences were not statistically significant (all *p* > 0.05) when examining the correlations between intimidation with various sociodemographic factors. Similar finding was observed after applying Benjamini–Hochberg FDR correction (Table 11). Effect sizes varied from 0.075 to 0.217, suggesting that the associations in Table 11 were predominantly weak, with just a few of them reaching moderate strength.

### 3.5. Distribution of Sociodemographic Among Participants Reporting Undermining Relationship Stability

Descriptive analyses of the participants reporting undermined relationship stability (n = 30, 20.5%) by various sociodemographic and clinical characteristics (Table 4) revealed that a considerable percentage had partners with low educational attainment (n = 18, 60%), while almost had a partner who was retired (n = 16, 53.3%). In addition, most of these participants had been married for >6 years (n = 28, 93.3%). Moreover, a considerable percentage of these participants were not the same age as their partners (n = 26, 86.7%). Furthermore, most of these participants had been diagnosed with breast cancer for >1 year (n = 22, 73.3%). Finally, most of these participants had undergone a mastectomy (n = 20, 66.7%; Table 12).

While there were variations in the proportions of specific groups, these differences were not statistically significant (all *p* > 0.05) when examining the associations between undermining relationship stability and various sociodemographic factors. Similar associations were found after applying Benjamini–Hochberg FDR correction. (Table 13). Effect sizes varied from 0.067 to 0.224, suggesting that the majority of the associations in Table 13 were weak, with a select few nearing moderate strength.

### 3.6. Interpretations of Directed Acyclic Graphs Related to Factors Contributing to Psychological Abuse

**Figure 1:**
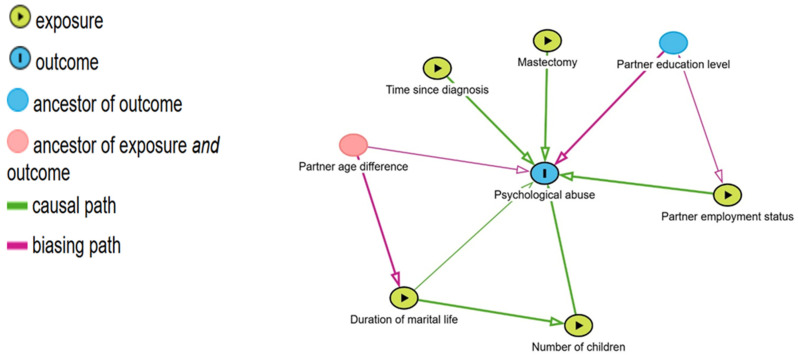
A Directed Acyclic Graph (DAG) of associations between sociodemographic, clinical, and partner-related variables and psychological abuse in breast cancer patients.

The DAG (Figure 1) aimed to conceptualize the hypothesized associations between sociodemographic, clinical, and partner-related factors in relation to psychological abuse. It was used to guide the specification and adjustment of the model, rather than to infer causality, due to the cross-sectional design.

The minimal sufficient adjustment set consisted of the partner employment status, the number of children, and the age difference between the partner and the participant, as indicated by the DAG. The graph shows that psychological abuse was statistically associated with partner education and employment status, while the duration of marriage and the number of children reflected interrelated familial and social dynamics. Clinical characteristics, such as time since diagnosis and mastectomy, were seen as correlates of psychological abuse rather than causal factors (Figure 1).

## 4. Discussion

A cancer diagnosis is a pivotal moment in the lives of patients and their families; it may incite familial discord and result in social, economic, and emotional ramifications [26]. Research indicates that a cancer diagnosis increases women’s susceptibility to abuse [27]. The literature on IPV among cancer survivors is relatively new and limited. Nonetheless, researchers are currently making strides in identifying and comprehending its effects on women who have been diagnosed with cancer. IPV among cancer survivors is only beginning to be investigated. The lack of research addressing this combined phenomenon has presented substantial obstacles for both researchers and practitioners as they strive to develop and implement prevention and intervention strategies. The most common form of IPV in Saudi Arabia and globally is psychological abuse [20,28]. Psychological violence is perpetrated chiefly through controlling conduct. Such behaviors include belittling, intimidation, threats of assault, insults, continual humiliation, and threatening to take away children [8,20]. The most common form of violence among women with breast cancer was psychological abuse, with the partner being the primary aggressor and the home being the most common location for the violence [8]. Therefore, this study aimed to investigate psychological abuse among patients with breast cancer and breast cancer survivors in Saudi Arabia, as well as the factors that are associated with this phenomenon. We propose that psychological abuse is prevalent among breast cancer patients and that its incidence is associated with specific socioeconomic factors. We found that among the 146 participants, 20.5% indicated they had experienced at least one form of psychological abuse. Low level of education, duration of marriage, age differences between spouses, and the experience of mastectomy were identified as primary variables contributing to psychological abuse among this population.

Among the Saudi women with breast cancer participating in our study, 20% reported experiencing insults, a decline in self-worth, and instability in their relationships with their partners; 17.8% reported experiences of belittlement and humiliation; and 15.1% reported they faced intimidation. This finding is consistent with a recent study in Iran reporting that women with breast cancer living with an abusive partner endure psychological abuse even during the treatment phase. This form of violence may be characterized by insulting language used by the intimate partner (verbal psychological violence), including curses and insults, or by abusive behaviors and body language (non-verbal psychological violence), such as sustained anger, refusal to communicate, or mistreatment [29]. These findings illustrate the psychological and emotional obstacles that women with breast cancer encounter in Saudi Arabia, stressing the detrimental influence on self-esteem and interpersonal relationships. Additionally, these findings highlight the importance of comprehensive support networks that address these persons’ physical and emotional well-being.

Descriptive analyses of various sociodemographic and clinical characteristics of our study participants also indicated that those who reported experiencing psychological abuse in any of its forms had partners with low educational levels and who were retired. This finding agrees with a previous study that reported that IPV was more likely to occur in Saudi Arabia when husbands had a low education level [20]. Additionally, most of our participants who had reported experiencing psychological abuse had been married for >6 years and were not the same age as their partners. A regional analysis from the Gulf Cooperation Council, including Saudi Arabia, revealed that the length of marriage can intensify IPV [30], which could explain the finding that women with breast cancer who have been married for >6 years are more likely to experience psychological abuse.

Other factors contributing to psychological abuse among our participants were breast cancer for >1 year and undergoing a mastectomy. Women who have undergone a mastectomy and those who have been diagnosed with breast cancer for an average of 5 years have encountered psychological abuse. It was observed that the intimate partners’ contempt, as well as expressions of humiliation, fear, and low self-esteem, reinforce the extent of psychological violence in the daily lives of women who have undergone mastectomies [31]. The partner is the most frequently cited aggressor [32]. This dynamic not only complicates the recuperation process but also impacts the mental health of breast cancer survivors. It is imperative to address these issues to ensure that these women receive comprehensive support and undergo both physical and emotional recovery.

We analyzed the correlations between abuse and various sociodemographic characteristics, such as age, partner education, partner work status, number of children, length of marriage, age difference, time since diagnosis, and mastectomy history. Although abuse appeared to be more common in some subgroups (e.g., a large number was among patients with lesser education vs. a low number among those with higher education), the majority of these differences were not statistically significant in chi-square (all *p* > 0.05). The lack of significance may represent a genuine lack of substantial associations between these features and psychological abuse, but it might also be attributed to the small number of patients who reported abuse (n = 30), which hampered the statistical power of our analysis. Similar limitations have been recognized in previous study of IPV with limited sample sizes, implying that larger investigations are required to reach more solid conclusions [33].

Nonetheless, some important associations were found. Patients whose spouses were unemployed or retired were more likely to report insults and belittlement, implying that financial difficulty or retirement-related stress can worsen marital conflict and abusive behaviors. During cancer treatment, financial strain, disruption of traditional domestic roles, and intensification of caregiving pressures may result from unemployment or retirement. These stressors have the potential to exacerbate tension and resentment within the relationship, thereby increasing the risk of psychological abuse. Patients with a partner age gap of ≥10 years and more than six children experienced belittlement more often. These findings may reflect unequal power dynamics in couples with significant age gaps, as well as the additional economic and caregiving demands of larger families. Although these relationships do not prove causality, they do identify potential demographic and relational risk variables that may be associated with psychological abuse in this population.

The DAG improved our analytical framework by elucidating potential confounding pathways and illustrating hypothesized associations among variables. This method reduced the likelihood of model misspecification or inappropriate adjustment and improved the transparency of the observed statistical relationships. Together, our findings highlight the complexities of psychological abuse among breast cancer patients. The limited number of significant correlations suggests that IPV is multifaceted, involving cultural, relational, and psychosocial elements not fully captured by demographic or clinical features.

While the study offers new insights, one should consider several limitations when interpreting the findings:

- Sampling bias: The survey was primarily distributed via social media platforms, potentially resulting in an underrepresentation of women over 50 and those with limited social media use. Further studies should employ probability or multi-center sampling to enhance representativeness.
- Self-reporting bias: Reliance on self-reporting may have introduced recall bias and social desirability bias. Mixed-method approaches, such as interviews, may yield better insights and validate self-reported data.
- Cross-sectional design: As this study is cross-sectional, causal inferences regarding the associations cannot be made. Longitudinal studies are suggested to elucidate temporal correlations.
- Small number of patients reporting abuse (n = 30): This small number reduced the statistical power of our analysis. Large samples are required to identify small effects.
- Limited power and Type II error risk: The likelihood of Type II error may have been elevated by the underachievement of the calculated sample size (n = 146), which implies that minor or moderate associations may have gone undetected. Subsequent investigations involving larger cohorts are necessary.
- Regression model constraints: The statistical power for the logistic regression may have been reduced due to the limited number of abuse cases, resulting in wide confidence intervals for multiple outcomes, especially for risk variables that were less prevalent. Studies that included more samples could model multiple IPV domains.
- Psychometric limitation: Given the study’s scope and sample size, additional construct validation (e.g., confirmatory factor analysis or known-groups validity) was not conducted, despite the adapted scale demonstrating satisfactory content validity and internal consistency. To improve the psychometric robustness of the instrument, future research should implement these methodologies.

Dichotomous response format: Binary response options limit the evaluation of severity gradients. Likert-based instruments may better capture a broader range of variation in future research.

Notwithstanding these limitations, our findings suggest several future directions to improve the recognition, prevention, and understanding of IPV. First, future studies should employ qualitative methods such as Interpretative Phenomenological Analysis to gain in-depth insights into the experiences of breast cancer patients and survivors in Saudi Arabia regarding IPV. This method will enable researchers to investigate the intricate personal and emotional narratives of these patients and survivors, thereby delivering a deeper understanding of their distinctive experiences. The results, focusing on personal narratives, may help shape more effective support systems and treatments customized to individuals’ specific needs. Second, IPV may be prevented by providing research grants to local investigators researching gender-based violence in chronic illness contexts. Third, it is crucial to implement awareness campaigns that educate husbands with low education levels about the importance of healthy relationships and nonviolent behavior. Fourth, Addressing IPV in breast cancer patients requires a multidisciplinary team effort. Therefore, it is essential to establish national protocols for healthcare professionals to recognize and address psychological abuse among patients with cancer. Standardized IPV screening protocols should be incorporated into oncology practice guidelines for ensuring the confidentiality and routine evaluation of psychological abuse. Incorporating these protocols into admission checklists, survivorship clinics, and psychosocial evaluations could facilitate early detection and prompt referral pathways. Oncology nurses, who frequently communicate with women diagnosed with breast cancer, are well-positioned to identify emotional warning signs and initiate secure, confidential conversations. It is imperative that clinical staff receive training in the identification of behavioral indicators, including but not limited to incoherent descriptions of distress, social withdrawal, reports of degrading partner behavior, and increased anxiety. Screening should be conducted in a private environment, with the patient alone, to guarantee safety and candid disclosure, as spouses may monitor or control clinical interactions [11]. Nurses should address concerns empathetically, and patients should be directed to referral resources such as hospital social workers, mental health services, advocates for victims of IPV, and national hotlines like the National Domestic Violence Hotline. A multidisciplinary approach is essential, with oncology nurses playing critical roles in establishing collaborative interventions that ensure trauma-informed, patient centered care addressing both illness and the unseen burden of violence. Fifth, to ensure that partners can provide supportive care without experiencing emotional strain, stakeholders should offer awareness and educational sessions. Finally, stakeholders, including the Family Affairs Council and the National Family Safety Program, may develop and implement educational and awareness campaigns to inform partners about preventing and reducing the prevalence of IPV in general and psychological abuse specifically, promoting it as a public concern [34].

## 5. Conclusions

Our findings offer insights into psychological abuse among patients with breast cancer in Saudi Arabia and its contributing factors. About one in five women with breast cancer in this study had experienced at least one form of psychological abuse. The greater incidence was in women whose partner was uneducated, unemployed, or retired. The length of marriage and duration of cancer also increased the risk of psychological abuse. Patients who had undergone mastectomy also reported increased vulnerability. These findings underscore the imperative of incorporating psychological abuse assessment into standard oncology practice, particularly for patients with identified risk factors, as IPV can complicate cancer treatment and management for women. It is important to strengthen collaboration between cancer and mental health services so that patients can be diagnosed early and receive the help they need.

## Figures and Tables

**Table 1:** The sociodemographic characteristics of the respondents (n = 146).

Demographics	n	%
**Age**		
Between the ages of 18 and 25	2	1.4
Between the ages of 26 and 35	12	8.2
Between 36 and 49 years	94	64
50 years of age or older	38	26
**Educational status**		
Secondary school diploma or less	72	49.3
Bachelor’s degrees	68	46.6
Master’s degree	4	2.7
Doctoral degree	2	1.4
**Partners’ education status**		
Secondary school diploma or less	90	61.6
Bachelor’s degrees	46	31.5
Master’s degree	6	4.1
Doctoral degree	4	2.7
**Occupation status**		
Employed	40	27.4
Retired	16	11
Unemployed	84	57.5
Working in business	6	4.1
**Partners’ occupation status**		
Employed	66	45.2
Retired	50	34.2
Unemployed	22	15.1
Working in business	8	5.5
**Relationship duration**		
Less than 3 years	8	5.4
Between 4 and 6 years	6	4.1
More than six years	132	90.4
**Partner age difference**		
Same age	34	23.3
5 to 9 years	48	32.9
Less than 5 years	40	27.4
More than 10 years	24	16.4
**Number of children**		
4–6 children	76	52.1
Three children or fewer	46	31.5
Six children or more	10	6.8
No children	14	9.6
**Duration of breast cancer**		
1–5 years	70	47.9
Less than a year	46	31.5
5–10 years	22	15.1
More than 10 years	8	5.4
**Mastectomy status**		
Undergo mastectomy	80	54.7
No undergo mastectomy	66	45.2

Note: Percentages are calculated from total respondents (n = 146). Values may not total 100% due to rounding.

**Table 2:** Psychometric properties of the IPV questions.

Variable	Corrected Item Total Correlation	Factor Loading
Did your partner insult you or make you feel an erosion of self-worth while you were sick with cancer?	0.92	0.85
Did your partner belittle and humiliate you while you were sick with cancer?	0.77	0.69
Did your partner use intimidation on you while you were sick with cancer?	0.81	0.69
Did your partner undermine relationship stability while you were sick with cancer?	0.70	0.57

Note: Psychometric properties are based on responses from the full sample (n = 146). Values represent corrected item–total correlations and standardized factor loadings from the one-factor solution.

**Table 3:** The frequency of psychological abuse among participants (n = 146).

Variable	Response	n	%
Did your partner insult you or make you feel an erosion of self-worth while you were sick with cancer?	Yes	30	20.5
Did your partner belittle and humiliate you while you were sick with cancer?	Yes	26	17.8
Did your partner use intimidation on you while you were sick with cancer?	Yes	22	15.1
Did your partner undermine relationship stability while you were sick with cancer?	Yes	30	20.5
≥1 form of abuse	Yes	30	20.5

Note: Percentages are calculated out of the total sample (n = 146). Values may not total 100% because participants could experience more than one form of psychological abuse.

**Table 4:** Characteristics of participants reporting insults and erosion of self-worth (n = 30).

Variable	n	%
**Patient age (years)**		
18–25	0	0.0
26–35	2	6.7
36–49	22	73.3
>50	6	20.0
**Partner education level**		
Secondary school or below	20	66.7
Bachelor’s degree	8	26.8
Master’s degree	0	0.0
Doctoral degree	2	6.7
**Partner occupation status**		
Unemployed	8	26.7
Employed	8	26.7
Retired	14	46.7
Business	0	0.0
**Duration of marital life (years)**		
<3	2	6.7
4–6	0	0.0
>6	28	93.3
**Partner age difference (years)**		
0 (same age)	6	20.0
<5	8	26.7
5–9	8	26.7
>10	8	26.7
**Number of children**		
0 (none)	4	13.3
≤3	12	40.0
4–6	10	33.3
>6	4	13.3
**Time since diagnosis (years)**		
<1	10	33.3
1–5	14	46.7
5–10	4	13.3
>10	2	6.7
**Mastectomy**		
No	12	40.0
Yes	18	60.0

Note: Percentages are calculated out of the subsample of participants who reported experiencing insults and erosion of self-worth (n = 30). Values may not total 100% due to rounding.

**Table 5:** Association between insults and erosion of self-worth and demographic characteristics among breast cancer patients (n = 146).

Variable	Yes n (%)	No n (%)	Test (*p*-Value)	FDR	Effect Size
**Patient age (years)**					
18–25	0 (0)	2 (100)			
26–35	2 (16.7)	10 (83.3)			
36–49	22 (23.4)	72 (76.6)	1.625		
>50	6 (15.8)	32 (84.2)	(0.801)	0.915	0.105
**Partner education level**					
Secondary school or below	20 (22.2)	70 (77.8)			
Bachelor’s degree	8 (17.4)	38 (82.6)			
Master’s degree	0 (0)	6 (100)	4.112		
Doctoral degree	2 (50.0)	2 (50.0)	(0.261)	0.693	0.168
**Partner occupation status**					
Unemployed	8 (36.4)	14 (63.6)			
Employed	8 (12.1)	58 (87.9)			
Retired	14 (28.0)	36 (72.0)	10.011		
Business	0 (0)	8 (100)	(0.017)	0.136	0.262
**Duration of marital life (years)**					
<3	2 (25.0)	6 (75.0)			
4–6	0 (0)	6 (100)	1.685		
>6	28 (21.2)	104 (78.8)	(0.503)	0.693	0.107
**Partner age difference (years)**					
0 (same age)	6 (17.6)	28 (82.4)			
<5	8 (20.0)	32 (80.0)			
5–9	8 (16.7)	40 (83.3)	3.029		
>10	8 (33.3)	16 (66.7)	(0.387)	0.693	0.144
**Number of children**					
0 (none)	4 (28.6)	10 (71.4)			
≤3	12 (26.1)	34 (73.9)			
4–6	10 (13.2)	77 (86.8)	6.277		
>6	4 (40.0)	6 (60.0)	(0.069)	0.276	0.207
**Time since diagnosis (years)**					
<1	10 (21.7)	36 (78.3)			
1–5	14 (20.0)	56 (80.0)			
5–10	4 (18.2)	18 (81.8)	0.225		
>10	2 (25.0)	6 (75.0)	(0.949)	0.949	0.039
**Mastectomy**					
No	12 (18.2)	54 (81.1)	0.413		
Yes	18 (22.5)	62 (77.5)	(0.520)	0.693	0.053

Note: Percentages are row percentages based on the total sample (n = 146). *p*-values were obtained using Pearson’s chi-square test, except when >20% of expected cell counts were < 5 or any cell contained 0, in which case Fisher’s Exact test was applied. Multiple-comparison correction was performed using the Benjamini–Hochberg False Discovery Rate (FDR) method. FDR = False Discovery Rate–adjusted *p*-values. Effect size for chi-square tests was reported using Cramer’s V. Values may not total 100% due to rounding.

**Table 6:** Characteristics of participants reporting belittlement and humiliation (n = 26).

Variable	n	%
**Patient age (years)**		
18–25	0	0.0
26–35	0	0.0
36–49	18	69.2
>50	8	30.8
**Partner education level**		
Secondary school or below	18	69.2
Bachelor’s degree	6	23.1
Master’s degree	0	0.0
Doctoral degree	2	7.7
**Partner occupation status**		
Unemployed	8	30.8
Employed	4	15.4
Retired	14	53.8
Business	0	0.0
**Duration of marital life (years)**		
<3	0	0.0
4–6	0	0.0
>6	26	100
**Partner age difference (years)**		
0 (same age)	4	15.4
<5	4	15.4
5–9	8	30.8
>10	10	38.5
**Number of children**		
0 (none)	2	7.7
≤3	10	38.5
4–6	8	30.5
>6	6	23.1
**Time since diagnosis (years)**		
<1	6	23.1
1–5	14	53.8
5–10	4	15.4
>10	2	7.7
**Mastectomy**		
No	8	30.8
Yes	18	69.2

Note: Percentages are calculated out of the subsample of participants who reported experiencing belittlement and humiliation (n = 26). Values may not total 100% due to rounding.

**Table 7:** Association between belittlement and humiliation and demographic characteristics among breast cancer patients (n = 146).

Variable	Yes n (%)	No n (%)	Test (*p*-Value)	FDR	Effect Size
**Patient age (years)**					
18–25	0 (0.0)	2 (100)			
26–35	0 (0.0)	12 (100)			
36–49	18 (19.1)	76 (80.9)	3.422		
>50	8 (21.1)	30 (78.9)	(0.350)	0.400	0.153
**Partner education level**					
Secondary school or below	18 (20.0)	72 (80.0)			
Bachelor’s degree	6 (13.0)	40 (88.0)			
Master’s degree	0 (0.0)	6 (100)	5.141		
Doctoral degree	2 (50.0)	2 (50.0)	(0.178)	0.285	0.188
**Partner occupation status**					
Unemployed	8 (36.4)	14 (63.6)			
Employed	4 (6.1)	62 (93.9)			
Retired	14 (28.0)	36 (72.0)	16.680		
Business	0 (0.0)	8 (100)	(<0.001)	0.008	0.338
**Duration of marital life (years)**					
<3	0 (0.0)	8 (100)			
4–6	0 (0.0)	6 (100)	3.355		
>6	26 (19.7)	106 (80.3)	(0.315)	0.420	0.152
**Partner age difference (years)**					
0 (same age)	4 (11.8)	30 (88.2)			
<5	4 (10.0)	36 (90.0)			
5–9	8 (16.7)	40 (83.3)	11.891		
>10	10 (41.7)	14 (58.3)	(0.008)	0.021	0.285
**Number of children**					
0 (none)	2 (14.3)	12 (85.7)			
≤3	10 (21.7)	36 (78.3)			
4–6	8 (10.5)	68 (89.5)	15.520		
>6	6 (60.0)	4 (40.0)	(0.003)	0.012	0.326
**Time since diagnosis (years)**					
<1	6 (13.0)	40 (87.0)			
1–5	14 (20.0)	56 (80.0)			
5–10	4 (18.2)	18 (81.8)	1.228		
>10	2 (25.0)	6 (75.0)	(0.718)	0.718	0.092
**Mastectomy**					
No	8 (12.1)	58 (87.9)	2.661		
Yes	18 (22.5)	62 (77.5)	(0.103)	0.206	0.135

Note: Percentages are row percentages based on the total sample (n = 146). *p*-values were obtained using Pearson’s chi-square test, except when >20% of expected cell counts were <5 or any cell contained 0, in which case Fisher’s Exact test was applied. Multiple-comparison correction was performed using the Benjamini–Hochberg False Discovery Rate (FDR) method. FDR = False Discovery Rate–adjusted *p*-values. Effect size for chi-square tests was reported using Cramer’s V. Values may not total 100% due to rounding.

**Table 8:** Multivariable logistic regression model predicting humiliation in participants with current or prior breast cancer (*n* = 146).

Predictor	B (SE)	Odds Ratio	95% CI for OR	*p*-Value
Partner employment status	1.68	5.36	1.62–17.74	0.006
Number of children >6 vs. ≤6	1.76	5.84	1.33–25.55	0.019
Partner age difference (years) >10 vs. ≤10	0.79	2.20	0.75–6.48	0.152

Note: Dependent variable = humiliation (yes vs. no). The table presents results from an exploratory logistic regression model including predictors that remained significant in bivariate analyses (collapsed model). Odds ratios (OR) are presented with 95% confidence intervals (CI). Values are based on complete cases (n = 146).

**Table 9:** Multivariable logistic regression model predicting belittlement/humiliation in participants with current or prior breast cancer (*n* = 146).

Predictor	B (SE)	Odds Ratio	95% CI for OR	*p*-Value	VIF
Partner employment status					
-Retired	1.56	4.74	1.39–16.17	0.013	1.03
-Unemployed	1.93	6.91	1.72–27.79	0.006	1.03
-Business/self-employed	−18.32	0.00	0.00	0.999	1.03
Number of children	0.20	1.23	0.69–2.19	0.493	1.00
Partner age difference (years)	0.34	1.40	0.87–2.24	0.165	1.03

Note: Dependent variable = belittlement/humiliation (yes/no). Estimates are from a multivariable logistic regression model. Odds ratios (ORs) are shown with 95% confidence intervals (CIs). Variance Inflation Factors (VIFs) ranged from 1.00 to 1.03 indicating no multicollinearity. Overall model performance was satisfactory (Nagelkerke R^2^ = 0.22; classification accuracy = 82.2%). Model fit indices: Hosmer–Lemeshow χ^2^ = 17.18, *p* = 0.028. A sensitivity analysis using Firth penalized logistic regression yielded similar ORs and *p* values.

**Table 10:** Characteristics of participants reporting intimidation (n = 22).

Variable	n	%
**Patient age (years)**		
18–25	0	0.0
26–35	2	9.1
36–49	18	81.8
>50	2	9.1
**Partner education level**		
Secondary school or below	16	72.7
Bachelor’s degree	4	18.2
Master’s degree	0	0.0
Doctoral degree	2	9.1
**Partner occupation status**		
Unemployed	6	27.3
Employed	7	27.3
Retired	10	45.5
Business	0	0.0
**Duration of marital life (years)**		
<3	2	9.1
4–6	0	0.0
>6	20	90.9
**Partner age difference (years)**		
0 (same age)	4	18.2
<5	8	36.4
5–9	6	27.3
>10	4	18.2
**Number of children**		
0 (none)	4	18.2
≤3	6	27.3
4–6	10	45.5
>6	2	9.1
**Time since diagnosis (years)**		
<1	6	27.3
1–5	10	45.5
5–10	4	18.2
>10	2	9.1
**Mastectomy**		
No	8	36.4
Yes	14	63.6

Note: Percentages are calculated out of the subsample of participants who reported experiencing intimidation (n = 22). Values may not total 100% due to rounding.

**Table 11:** Association between intimidation and demographic characteristics among breast cancer patients (n = 146).

Variable	Yes n (%)	No n (%)	Test (*p*-Value)	FDR	Effect Size
**Patient age (years)**					
18–25	0 (0.0)	2 (100)			
26–35	2 (16.8)	10 (83.3)			
36–49	18 (19.1)	76 (80.9)	4.456		
>50	2 (5.3)	36 (94.7)	(0.169)	0.451	0.175
**Partner education level**					
Secondary school or below	16 (17.8)	74 (82.2)			
Bachelor’s degree	4 (8.7)	42 (91.3)			
Master’s degree	0 (0.0)	6 (100)	6.854		
Doctoral degree	2 (50.0)	2 (50.0)	(0.089)	0.356	0.217
**Partner occupation status**					
Unemployed	6 (27.3)	16 (72.7)			
Employed	6 (9.1)	60 (90.9)			
Retired	10 (20.0)	40 (80.0)	6.773		
Business	0 (0.0)	8 (100)	(0.088)	0.704	0.215
**Duration of marital life (years)**					
<3	2 (25.0)	6 (75.0)			
4–6	0 (0.0)	6 (100)	1.682		
>6	20 (15.2)	112 (84.8)	(0.489)	0652	0.107
**Partner age difference (years)**					
0 (same age)	4 (11.8)	30 (88.2)			
<5	8 (20.0)	32 (80.0)			
5–9	6 (12.5)	42 (87.5)	1.345		
>10	4 (16.7)	20 (83.3)	(0.718)	0.821	0.096
**Number of children**					
0 (none)	4 (28.6)	10 (71.4)			
≤3	6 (13.0)	40 (87.0)			
4–6	10 (13.2)	66 (86.8)	2.549		
>6	2 (20.0)	8 (80.0)	(0.414)	0.663	0.132
**Time since diagnosis (years)**					
<1	6 (13.0)	40 (87.0)			
1–5	10 (14.3)	60 (85.7)			
5–10	4 (18.2)	18 (81.8)	0.964		
>10	2 (25.0)	6 (75.0)	(0.729)	0.729	0.081
**Mastectomy**					
No	8 (12.1)	58 (87.9)	0.818		
Yes	14 (17.5)	66 (82.5)	(0.366)	0.732	0.075

Note: Percentages are row percentages based on the total sample (n = 146). *p*-values were obtained using Pearson’s chi-square test, except when >20% of expected cell counts were < 5 or any cell contained 0, in which case Fisher’s Exact test was applied. Multiple-comparison correction was performed using the Benjamini–Hochberg False Discovery Rate (FDR) method. FDR = False Discovery Rate–adjusted *p*-values. Effect size for chi-square tests was reported using Cramer’s V. Values may not total 100% due to rounding.

**Table 12:** Characteristics of participants reporting undermining relationship stability (n = 30).

Variable	n	%
**Patient age (years)**		
18–25	0	0.0
26–35	2	6.7
36–49	20	66.7
>50	8	26.7
**Partner education level**		
Secondary school or below	18	60.0
Bachelor’s degree	10	33.3
Master’s degree	0	0.0
Doctoral degree	2	6.7
**Partner occupational status**		
Unemployed	4	13.3
Employed	10	33.3
Retired	16	53.3
Business	0	0.0
**Duration of marital life (years)**		
<3	2	6.7
4–6	0	0.0
>6	28	93.3
**Partner age difference (years)**		
0 (same age)	4	13.3
<5	12	40.0
5–9	8	26.7
>10	6	20.0
**Number of children**		
0 (none)	2	6.7
≤3	10	33.3
4–6	14	46.7
>6	4	13.3
**Time since diagnosis (years)**		
<1	8	26.7
1–5	14	46.7
5–10	4	13.3
>10	4	13.3
**Mastectomy**		
No	10	33.3
Yes	20	66.7

Note: Percentages are calculated out of the subsample of participants who reported experiencing undermining relationship stability (n = 30). Values may not total 100% due to rounding.

**Table 13:** Association between undermining relationship stability and demographic characteristics among breast cancer patients (n = 146).

Variable	Yes n (%)	No n (%)	Test (*p*-Value)	FDR	Effect Size
**Patient age (years)**					
18–25	0 (0.0)	2 (100)			
26–35	2 (16.8)	10 (83.3)			
36–49	20 (21.3)	74 (78.7)	0.664		
>50	8 (21.1)	30 (78.9)	(1.000)	1.000	0.067
**Partner education level**					
Secondary school or below	18 (20.0)	72 (80.0)			
Bachelor’s degree	10 (21.7)	36 (78.3)			
Master’s degree	0 (0.0)	6 (100)	3.734		
Doctoral degree	2 (50.0)	2 (50.0)	(0.315)	0.504	0.160
**Partner occupation status**					
Unemployed	4 (18.2)	18 (81.8)			
Employed	10 (15.2)	56 (84.8)			
Retired	16 (32.0)	34 (68.0)	7.338		
Business	0 (0.0)	8 (100)	(0.074)	0.592	0.224
**Duration of marital life (years)**					
<3	2 (25.0)	6 (75.0)			
4–6	0 (0.0)	6 (100)	1.685		
>6	28 (21.2)	104 (78.8)	(0.503)	0.575	0.107
**Partner age difference (years)**					
0 (same age)	4 (11.8)	30 (88.2)			
<5	12 (30.0)	28 (70.0)			
5–9	8 (16.7)	40 (83.3)	4.530		
>10	6 (25.0)	18 (75.0)	(0.210)	0.560	0.176
**Number of children**					
0 (none)	2 (14.3)	12 (85.7)			
≤3	10 (21.7)	36 (78.3)			
4–6	14 (18.4)	62 (81.6)	2.905		
>6	4 (40.0)	6 (60.0)	(0.420)	0.560	0.141
**Time since diagnosis (years)**					
<1	8 (17.4)	38 (82.6)			
1–5	14 (20.0)	56 (80.0)			
5–10	4 (18.2)	18 (81.8)	4.620		
>10	4 (50.0)	4 (50.0)	(0.244)	0.488	0.178
**Mastectomy**					
No	10 (15.2)	56 (84.8)	2.149		
Yes	20 (25.0)	60 (75.0)	(0.143)	0.572	0.121

Note: Percentages are row percentages based on the total sample (n = 146). *p*-values were obtained using Pearson’s chi-square test, except when >20% of expected cell counts were < 5 or any cell contained 0, in which case Fisher’s Exact test was applied. Multiple-comparison correction was performed using the Benjamini–Hochberg False Discovery Rate (FDR) method. FDR = False Discovery Rate–adjusted *p*-values. Effect size for chi-square tests was reported using Cramer’s V. Values may not total 100% due to rounding.

## Data Availability

The data are not publicly available due to privacy and ethical restrictions. Anonymized data may be shared upon rea-sonable request and with prior approval from the Ethics Committee of Umm Al-Qura University.
